# Development of single nucleotide polymorphisms in key genes of taurine and betaine metabolism in Crassostrea hongkongensis and their association with content-related traits

**DOI:** 10.21203/rs.3.rs-5097219/v1

**Published:** 2024-12-18

**Authors:** Lingxin Kong, Ziao Chen, Zhen Jia, Qiong Deng, Peng Zhu, Youhou Xu, Zhicai She

**Affiliations:** Beibu Gulf University; Beibu Gulf University; Beibu Gulf University; Beibu Gulf University; Beibu Gulf University; Beibu Gulf University; Beibu Gulf University

**Keywords:** Crassostrea hongkongensis, taurine, betaine, SNP, association analysis

## Abstract

**Background:**

Taurine and betaine are important nutrients in *Crassostrea hongkongensis* and have many important biological properties. To investigate the characteristics of taurine and betaine contents and identify SNPs associated with traits in the *C.hongkongensis*, we cloned the full-length cDNA of key genes in taurine and betaine (unpublished data) metabolism, determined taurine and betaine content and gene expression in different tissues and months of specimen collection, and developed SNPs in the gene coding region.

**Results:**

We cloned the full-length cDNA of cysteine dioxygenase (*ChCDO*) and cysteine sulfite decarboxylase (*ChCSAD*), which are key genes involved in taurine metabolism in *C. hongkongensis*, and found that betaine and taurine contents and the expression of key genes were regulated by seawater salinity. A total of 47 SNP markers were developed in the coding regions of *ChCSAD*, *ChCDO*, *ChCDH*, *ChBADH*, and *ChBHMT* using gene fragment resequencing and FLDAS-PCR. Through association analysis in a population of *C. hongkongensis* in the Maowei Sea, Guangxi, nine SNPs were found to be associated with taurine content, and one SNP was associated with betaine content. Haploid and linkage disequilibrium analyses showed that SNPs in *ChCDO* formed one linkage group with three haplotypes: ACACA, GTTTG, and GTACA. The average taurine content of the corresponding individuals was 873.88, 838.99, and 930.72 ng/g, respectively, indicating the GTACA haplotype has a significant advantage in terms of taurine content.

**Conclusions:**

We identified SNPs associated with taurine and betaine contents in *C.hongkongensis* for the first time, and found the GTACA haplotype in the *ChCDO* coding region has a significant advantage in taurine content. These loci and haplotypes can serve as potential molecular markers for the molecular breeding of *C. hongkongensis*.

## Background

With the development of the economy and deepening of people’s health awareness, an increasing number of people choose seafood with higher nutritional content [1, 2]. Taurine and betaine are important nutrients in *C. hongkongensis* and have many important biological properties [3, 4]. Oysters are also one of the most abundant natural sources of taurine. Since the beginning of the 21st century, oyster production in China has increased significantly. In 2019, China’s oyster farming production reached 5.2 million tons, of which *C. hongkongensis* represented approximately 1.8 million, accounting for 34.45% of China’s total oyster production [5]. Although the production of *C. hongkongensis* is high, it is still difficult to meet the market demand for high-quality oysters, and the economic benefits they bring are relatively low. The reasons behind this are factors such as excessive aquaculture in the sea and changes in the aquaculture environment, which have led to a decline in the quality of *C. hongkongensis*.

Betaine and taurine not only serve as important nutrients in oysters but also give them a unique freshness quality [6, 7]. With the continuous increase in research on betaine and taurine, they have been found to buffer substances for osmotic pressure fluctuations, which can improve the tolerance of biological cells to high-salt and high-osmotic environments [8, 9]. Pierce et al. [10] found significant differences in the betaine synthesis rates among populations of *Crassostrea virginica* under different salinities. The betaine synthesis rate of populations adapted to high salinities was 3–4 times greater than that of populations adapted to low salinity. Song et al. [11] also found that taurine content in the adductor muscle of *C. hongkongensis* changed significantly with salinity. When *C. hongkongensis* was moved to 30‰ seawater, taurine content increased rapidly; however, 24 h after moving to 6‰ seawater, taurine content decreased by approximately 40%.

Cysteine dioxygenase (CDO) and cysteine sulfinic acid decarboxylase (CSAD) are key enzymes involved in taurine synthesis [12]. Marine bivalves mainly use cysteine as a raw material to maintain taurine content in their bodies [13]. Cysteine is oxidized to cysteine sulfite (CSA) through CDO and decarboxylated to hypotaurine through CSAD. Finally, hypotaurine is oxidized to taurine. In marine mollusks, full-length cDNA sequences of CDO and CSAD genes have been cloned from *Crassostrea gigas* and *Bathymodiolus septemdierum*, and their expression levels have been found to be related to changes in taurine content [14, 15]. Choline dehydrogenase (CDH) and betaine aldehyde dehydrogenase (BADH) have long been considered important enzymes for catalyzing the production of betaine. Betaine is generated from choline through two enzymatic reactions. First, choline is catalyzed by CDH to form betaine aldehyde, which is then oxidized to betaine by BADH [16, 17]. Betaine homocysteine methyltransferase (BHMT) is a key enzyme in betaine catabolism [18]. Multiple metabolic pathways are involved in the decomposition of betaine in living organisms, the most common being the methionine cycle, which is catalyzed by BHMT [19]. Previous studies have shown that betaine synthesis in *Litopenaeus vannamei* and *Chasmagnathus granulata* depends on the activities of CDH and BADH [20, 21], and the expression of *BHMT* was found to affect the betaine content in *C. gigas* [22]. To date, there have been no reports on the key genes involved in taurine and betaine metabolism in *C. hongkongensis*.

To explore the relationship between key genes related to taurine and betaine metabolism and content-related traits in *C. hongkongensis*, we determined taurine and betaine contents, as well as gene expression of *ChCDO*, *ChCSAD*, *ChBADH*, *ChCDH*, and *ChBHMT* in different tissues and specimen collection months. Then, we conducted an association analysis to screen single-nucleotide polymorphism (SNP) loci associated with taurine and betaine contents, laying a foundation for the breeding of high-quality oysters.

## Methods

### Oyster collection

Oysters used for analysis at different times and in different tissues were obtained from the Sandun Aquaculture Area in Qinzhou, Guangxi, China. Oysters for analysis at different times were collected once a month from June 2020 to April 2021. Oysters were collected from the same location, and 15 individuals were collected each time. Adductor muscles, gills, and other tissues were separated, frozen in liquid nitrogen, and stored at − 80°C for taurine and betaine content detection and RNA extraction. Samples from April were used for analysis of different tissues, including the adductor muscle, gills, mantle, gonads, digestive glands, and labial palps.

One hundred and five oysters used for SNP development and association analysis were collected from Maowei Sea. The oysters were attached at the same time, and grew and developed in the same natural environment. The adductor muscle, gills, and other tissues were separated, frozen in liquid nitrogen, and stored at − 80°C for taurine and betaine content detection and RNA extraction.

### Full-length cDNA cloning of key genes

Total RNA was extracted from the samples using the TRIzol method. The extracted RNA was measured by spectrophotometry with a nucleic acid protein concentration meter (Gene Company Limited, Thermo, China). RNA integrity was detected by 1% agarose gel electrophoresis, and RNA was reverse transcribed into cDNA with a TransGen Biotech reverse transcription kit. Primers were designed for intermediate fragment cloning using the CSAD and CDO sequences of *C. gigas* as references. Using cDNA as a template, 3’ and 5’ specific primers were designed in the cloned intermediate fragments for 3’ and 5’ RACE amplification (Table 1). The product was detected by 1% agarose gel electrophoresis, connected to the PMD19 clone vector and transformed into T1 *Escherichia coli* cells. After overnight cultivation and PCR detection, the bacterial suspension was sent to Shanghai Shenggong Biotechnology Co., Ltd. for sequencing.

### Gene sequence and evolutionary analysis

Utilizing ORF Finder online tools (https://www.ncbi.nlm.nih.gov), we predicted the open reading frame (ORF) and amino acid sequences based on the full-length cDNA sequence. We predicted the molecular weight, isoelectric point, hydrophobicity, signal peptide, and structural domain of proteins using online software such as Expasy (https://web.expasy.org), Signalp (http://www.cbs.dtu.dk/services/SignalP/), and NCBI. After using the BLAST tool in the NCBI database for sequence alignment and searching for homologous protein sequences, we performed multiple comparisons of homologous sequences using DNAMAN 7.0 software and built an evolutionary tree using MEGA 5.1 software.

### Analysis of taurine and betaine content

Taurine and betaine contents were detected using taurine and betaine enzyme-linked immunosorbent assay (ELISA) kits (Shanghai Meilian Biotechnology Co., Ltd., Shanghai, China). Blank, standard, and sample wells were included in testing. We added 50 μL of standard solution into each standard well, and then introduced sample diluent (40 μL) and sample solution (10 μL) to each sample well, followed by incubation at 37 °C for 30 min and five washes. Next, we added 50 μL of HRP enzyme-linked immunosorbent assay reagent to each well and 100 μL of acidic chromogenic agent for color development for 10 min at 37 °C in the dark. Absorbances were measured at 450 nm using an ELISA reader (Molecular Devices, Spectra Max Id5, USA), and the nutrient contents were calculated using a standard curve. Statistical analysis was performed using SPSS 22.0. Variance analysis was performed for samples collected at different times and from different tissues, and descriptive statistical analysis was performed for samples used for association analysis.

### Gene expression analysis in different tissues and at different times

Total RNA was extracted from gills collected in different months and various tissue samples from April using the TRIzol method. The RNA was reverse transcribed into cDNA and diluted 10 times to serve as a quantitative PCR template (Table 1). Then, qRT-PCR was performed using a fluorescence quantitative PCR instrument (Bio-Rad, CFX96, USA). Each experimental group included three biological replicates and three technical replicates. L-Actin served as a reference gene. The relative expression level of the target gene was calculated using the 2^−△△CT^ method, with results expressed as the mean ± standard deviation (SD). Analyses of variance (ANOVA) and multiple comparison testing was performed using SPSS 22.0 software.

### Development of SNPs

Using the full-length cDNA of *ChCSAD*, *ChCDO*, *ChBADH*, *ChCDH*, and *ChBHMT* genes as templates, Oligo 7 was used to design primers for amplifying the ORF region of genes (Table S1). Ten oysters with significant differences in taurine or betaine contents were selected to clone the same gene fragment, and the PCR products were sent to Shanghai Shenggong Biotechnology Co., Ltd., for sequencing. We then used DNAMAN sequence comparison to predict candidate SNPs, followed by fragment length discrepant allele specific PCR (FLDAS-PCR) and polyacrylamide gel electrophoresis for SNP validation. First, we designed two upstream primers of different lengths, each with a 3’ end that paired with two SNP allele bases, using Oligo 7. At the same time, we introduced mismatches at the 3rd or 4th base of the 3’ end of the two allele-specific primers to increase specificity. Then, we added base sequences of different lengths at the 5’ end, downstream was a universal primer sequence. The lengths of the amplified DNA fragments were 100–200 bp. The amplified product was detected by 12% polyacrylamide gel electrophoresis.

### Population genotyping

FLDAS-PCR and polyacrylamide gel electrophoresis were used for genotyping when the SNP density was greater than 1/20 bp. Owing to the different lengths of the upstream primers, the homozygotes were shown as a single band, and the heterozygotes as two bands with a size difference of 8 bp.

Gene fragment resequencing was used for genotyping when SNP density was less than 1/20 bp. First, the target fragments of all samples were cloned, and the PCR products were recovered, purified, and sent to Shanghai Shenggong Biotechnology Co., Ltd., for sequencing. We then compared the sequencing peaks of the same target fragment from different samples and genotyped the candidate SNPs in the population.

### Association analysis

Using SPSS 22.0, an association analysis between the content of taurine and betaine and genotype data was conducted using one-way ANOVA or t-testing. SNP markers that showed significant differences between different genotypes were considered associated with taurine or betaine content.

### Haploid and linkage analysis

We used Haploview 4.2 to perform haplotype and linkage analyses for the SNPs considered significant from the association analysis and used ANOVA in SPSS 22.0 to statistically analyze the taurine and betaine content between individuals with different haplotypes.

## Results

### Characteristics of full-length cDNA of ChCSAD and ChCDO

The total length of *ChCSAD* cDNA was 2140 bp (NCBI login number: OP792983), including a 55-bp 5’ untranslated region (UTR), 417-bp 3’ UTR, and 668-bp ORF, encoding a total of 555 amino acids (Fig. S1). The relative molecular weight of the protein was 63.45 KDa, and its isoelectric point was 7.60. The *ChCSAD* protein was predicted to contain a conserved pyridoxal-deC domain (113–479) (Fig. S2). The total length of the *ChCDO* cDNA was 1402 bp (NCBI login number: OP792984), including a 5’ UTR of 196 bp, 3’ UTR of 705 bp, and ORF of 501 bp, encoding a total of 166 amino acids (Fig. S3). The relative molecular weight of the protein was 19.04 KDa, with an isoelectric point of 6.41. The predicted conserved domain in the *ChCDO* protein was a CDOI domain (1–140) (Fig. S4)

### Homology and phylogenetic analysis of ChCSAD and ChCDO

**Figure 1:**
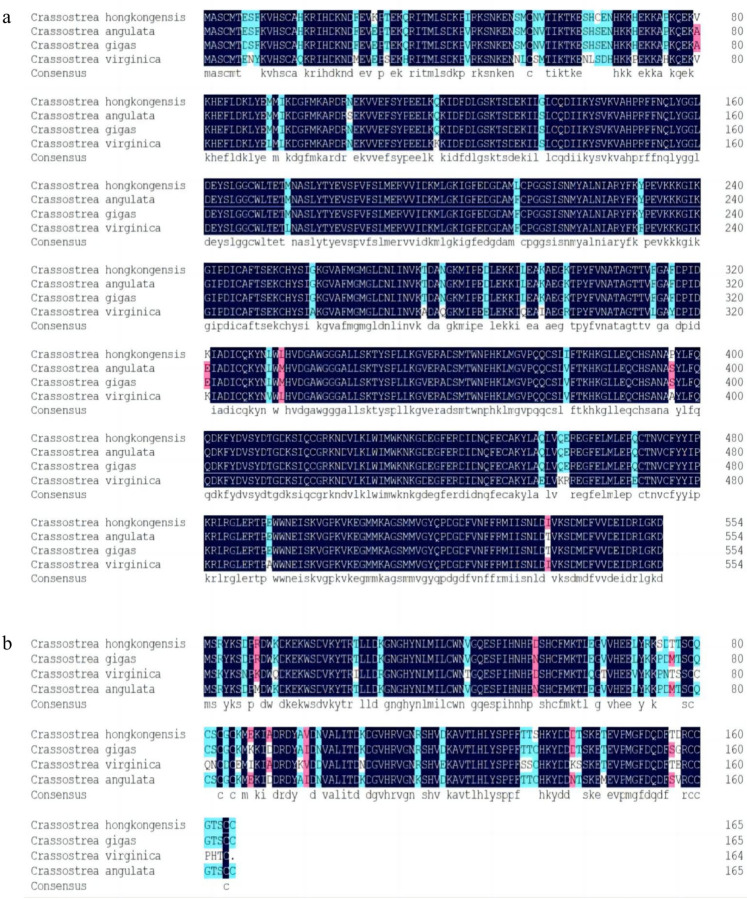
Multiple sequence alignments of ChCSAD (A) and ChCDO (B) Black: domains with high similarity; blue: domains with lower similarity; red: domains with relatively high similarity.

**Figure 2:**
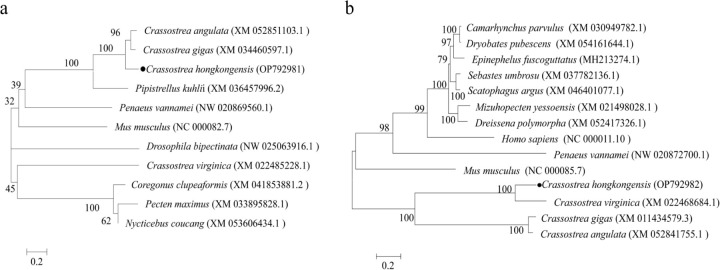
Phylogenetic analysis of ChCSAD (A) and ChCDO (B) The number next to the internal branch represents the value based on 1000 repeated calculations, and 0.2 represents the genetic distance.

Alignment of the amino acid sequences of ChGSAD and ChCDO in *C. hongkongensis* with those of *C. gigas*, *C. virginica*, and *C. angulata* revealed high amino acid conservation (Fig. 1). The phylogenetic tree showed that ChCSAD and ChCDO in *C. hongkongensis* were closely related to those in other invertebrates such as *C. gigas* and *C. angulata* (Fig. 2).

### Betaine and taurine contents and key gene expression in different tissues

**Figure 3:**
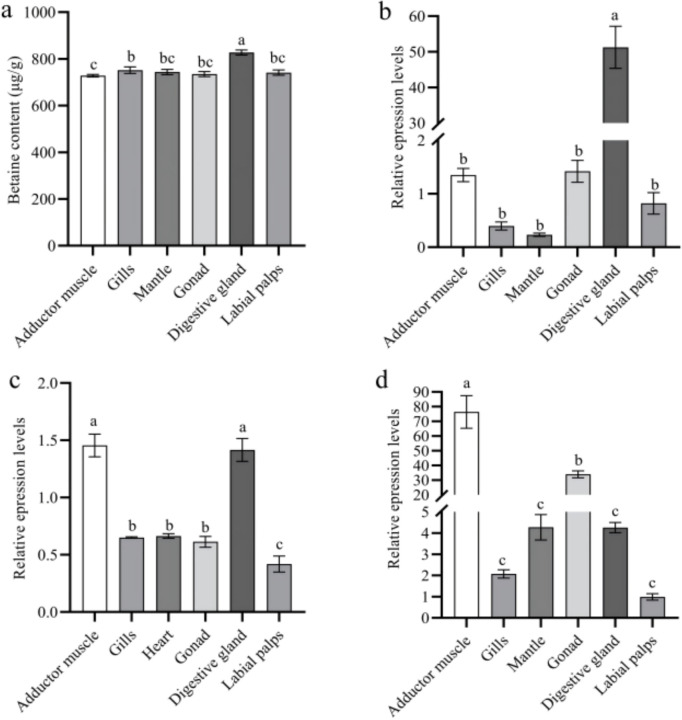
Betaine contents and gene expression in different tissues a, Betaine contents in different tissues of*C. hongkongensis*; b, *ChBHMT*expression in different tissues; c, *ChCDH* expression in different tissues; d, *ChBADH* expression in different tissues. Different letters indicate significant differences (*P* < 0.05).

**Figure 4:**
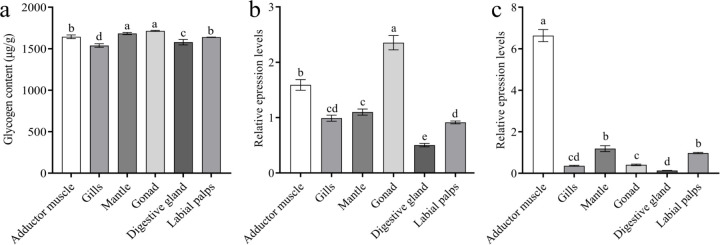
Taurine contents and gene expression in different tissues a, Taurine contents in different tissues of*C. hongkongensis*; b, *ChCSAD* expression in different tissues; c, *ChCDO* expression in different tissues. Different letters indicate significant differences (*P* < 0.05).

The highest betaine content in *C. hongkongensis* was found in the digestive gland (827.51 μg/g), and this amount was significantly higher than that in other tissues (*P* < 0.05) (Fig. 3a). *ChCDH* and *ChBADH* were expressed at the highest levels in the adductor muscle (*P* < 0.05), whereas *ChBHMT* was expressed in the digestive gland (*P* < 0.05) (Fig. 3b–d). Taurine content was the highest in the digestive gland (970.81 μg/g), and this amount was significantly higher than that in other tissues (*P* < 0.05) (Fig. 4a). The expression of *ChCSAD* reached its highest level in the digestive gland (*P* < 0.05), whereas *ChCDO* was highest in the adductor muscle and digestive gland (*P* < 0.05) (Fig. 4b–c).

### Betaine and taurine contents and key gene expression in different months

**Figure 5:**
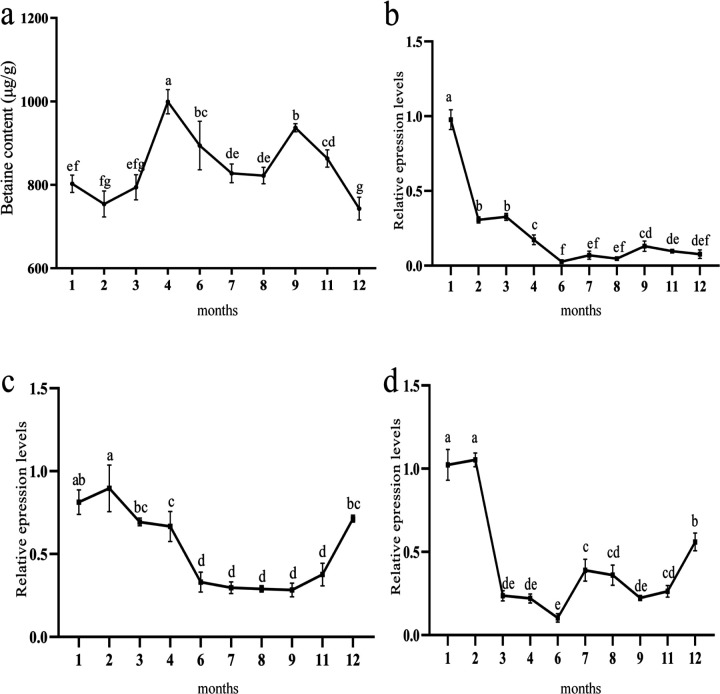
Betaine contents and gene expression in different months a, Betaine contents in *C.hongkongensis* in different months; b, *ChBHMT* expression in different months; c, *ChCDH* expression in different months; d, *ChBADH* expression in different months. Different letters indicate significant differences (*P* < 0.05).

Betaine content in *C. hongkongensis* steadily increased from 754.08 μg/g in February to a peak of 999.25 μg/g in April, then rapidly dropped to the lowest level of 743.01 μg/g in December (Fig. 5a). Expression of *ChCDH* was high from January to February, significantly decreased from February to March and remained low from March to November. Expression of *ChBADH* was high from January to April, decreased significantly from April to June and remained low from June to November. *ChBHMT* expression was significantly higher in January than in the other months (*P* < 0.05) and remained low after a significant decrease in February (Fig. 5b–d).

**Figure 6:**
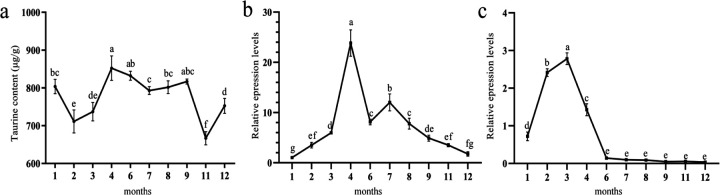
Taurine contents and gene expression in different months a, Taurine content in *C. hongkongensis* in different months; b, *ChCSAD* expression in different months; c, *ChCDO* expression in different months. Different letters indicate significant differences (*P* < 0.05).

Taurine and betaine contents showed a similar trend, with taurine content increasing from February to April and reaching its highest value of 852.32 μg/g in April. From June to December, taurine showed a decreasing trend and dropped to its lowest value of 666.75 μg/g in November (Fig. 6a). *ChCSAD* and *ChCDO* expression levels gradually increased from January, reaching their highest levels in April and March, respectively, and then gradually decreased (*P* < 0.05) (Fig. 6b–c).

### Characteristics of betaine and taurine content in association analysis population

The taurine content of the experimental individuals ranged from 779.46-957.30 μg/g (Fig. S5a), with an average of 862.95 ± 51.42 μg/g and a coefficient of variation (CV) of 5.96%; The betaine content of the experimental individuals ranged from 809.69-1036.57 μg/g (Fig. S5b), with an average of 911.02 ± 67.18 μg/g and a coefficient of variation (CV) of 7.37%. There is significant variation in the content of taurine and betaine, which follows a normal distribution (*P > 0.05*) and is suitable for conducting association analysis.

### SNPs in the coding regions of key genes

**Figure 7:**
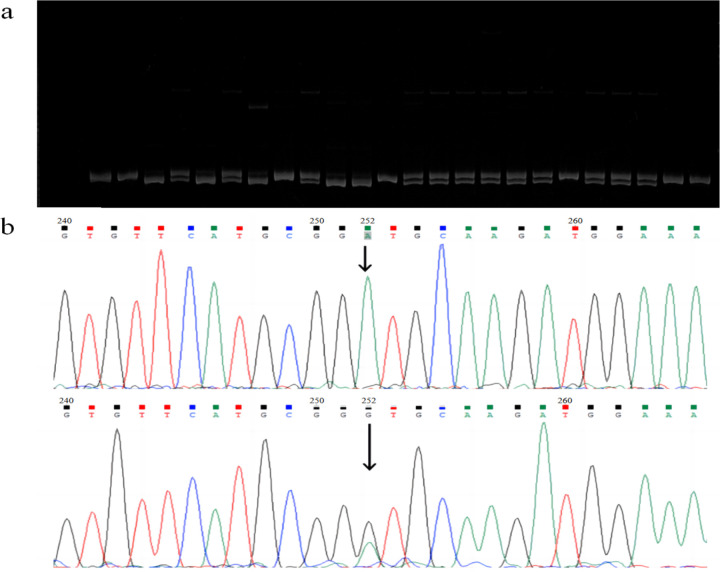
SNP genotyping results a, SNP genotyping results based on FLDAS-PCR of the three different genotypes shown; two bands indicate heterozygous and one band indicates homozygous; b, SNP genotyping results based on gene fragment resequencing for nucleotide site 252 indicated by the arrow; the single peak in the figure above indicates homozygous, and the double peaks in the figure below indicate heterozygous.

Using gene fragment cloning, Sanger sequencing, and multi-sequence alignment, 107 candidate SNPs were predicted for *ChCSAD*, *ChCDO*, *ChBADH*, *ChCDH*, and *ChBHMT*. A total of 107 sets of primers were designed, and 47 SNP loci were validated, all of which were successfully genotyped in the experimental population (Fig. 7). The number of SNPs for each gene is shown in Table S2, and information on the 47 validated SNP loci is shown in Table S3.

### SNPs associated with betaine and taurine contents

Nine SNPs were significantly correlated with taurine content (*P* < 0.05), and one SNP was significantly correlated with betaine content (*P* < 0.05) (Table 2). Among them, three SNPs caused coding amino acid changes. SNP *ChCDO*-46 changed lysine (Lys) to glutamate (Glu), *ChCDO*-394 changed threonine (Thr) to serine (Ser), and *ChCDH*-1585 changed asparagine (Asn) to aspartate (Asp) (Table 3).

### Haplotype and linkage analysis

**Figure 8:**
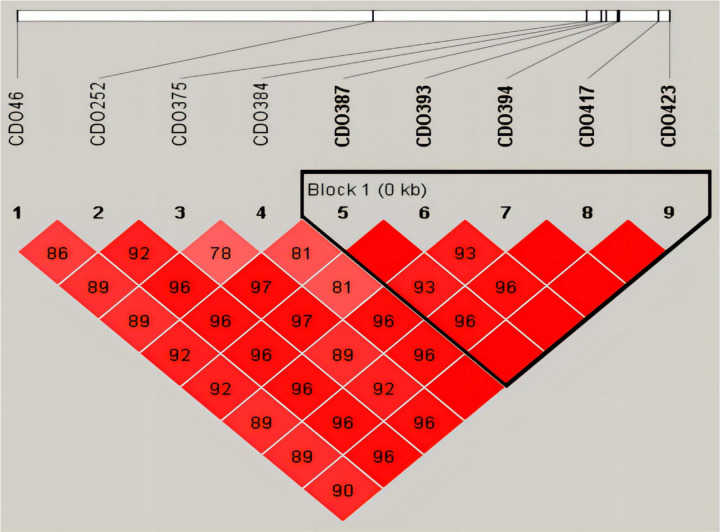
Linkage disequilibrium (LD) for the nine SNPs associated with taurine SNP IDs are shown in the upper panels. D’ > 80 was the threshold of linkage disequilibrium. Darker colors indicate stronger linkage disequilibrium of SNPs.

Among the nine SNPs associated with taurine content, five (*ChCDO*-387, *ChCDO*-393, *ChCDO*-394, *ChCDO*-417, *ChCDO*-423) formed a linkage group and three haplotypes (Fig. 8), ACACA, GTTTG, and GTACA, with haplotype frequencies of 0.781, 0.176, and 0.033, respectively. Among them, the GTACA haplotype had a significant advantage in taurine content (Table 4).

## Discussion

### Characteristics of betaine and taurine content and key gene expression in C. hongkongensis

In this study, we found that betaine content in the digestive glands of *C. hongkongensis* was the highest among all tissues (*P* < 0.05). We speculate that the digestive gland is the energy and fat storage organ, as well as food digestive organ, and the main site of betaine synthesis in oysters [23]. *ChCDH* and *ChBHMT* are highly expressed in the digestive gland and adductor muscles, whereas *ChBADH* is highly expressed in adductor muscles. The adductor muscle is an important component of oyster muscles, and it plays an important role in energy storage, metabolism, and other life processes. The adductor muscle of purple-shelled clams synthesizes betaine from choline, and we speculate that it has the same function as the oyster adductor muscle [24]. As a filter-feeding animal, *C. hongkongensis* mainly filters and feeds on planktonic algae and organic debris to meet its energy needs. The digestive gland is an important organ that participates in oyster lipid and carbohydrate metabolism [25].

The highest taurine content was found in the digestive gland, suggesting that the digestive glands of oysters have functions similar to those of the livers of higher animals, and that it is the main organ for synthesizing taurine [26]. *ChCSAD* is expressed most highly in the digestive gland, suggesting that *ChCSAD* is a key gene involved in the synthesis of taurine in oysters, and that the digestive gland is the main organ for taurine synthesis in oysters. Chen et al. [27] found that CSAD was widely expressed in all tissues of razor clams, with the highest expression in the liver. *ChCDO* is highly expressed in the adductor muscle, possibly due to its indirect involvement in the regulation of taurine osmotic pressure in oysters, with the adductor muscle serving as the main site for taurine metabolism and accumulation during salinity adaptation. As early as 1966, Lynch [28] found significant changes in the contents of taurine, alanine, glycine, and proline in the shell-closing muscle of American oysters with changes in salinity.

### Factors affecting betaine and taurine content and gene expression in C. hongkongensis

Betaine is a buffering substance for osmotic pressure fluctuations that can enhance the tolerance of biological cells to high salt and high osmotic environments and increase the tolerance of aquatic animals to osmotic pressure fluctuations [29–31]. Free amino acids (FAA) have also been shown to play a major role in regulating intracellular osmotic pressure and cell volume [32]. Taurine, one of the main free amino acids in shellfish, plays an important role in maintaining osmotic pressure in oysters. When salinity decreases, oysters release large amounts of taurine to adapt to low-osmotic environments. Conversely, taurine accumulates in and adapts to highly osmotic environments [33]. In our study, we found that the levels of betaine and taurine in *C. hongkongensis* reached their highest levels in April, possibly because of an increase in salinity caused by high temperatures and low rainfall in Guangxi in April. Taurine and betaine contents showed a downward trend from June to December, which may have been due to increased rainfall after May that continued until October, resulting in a decrease in seawater salinity. Lin et al. [34] also found that betaine content in oysters changes when exposed to changes in external salinity. Taurine also participates in osmotic regulation to reduce the damage to oysters caused by changes in environmental salinity. Huang et al. [35] found that the taurine content of oysters in high-salinity waters was higher than that in low-salinity waters. The present study also found that the levels of betaine and taurine in oysters changed with changes in seawater salinity, further indicating that betaine and taurine may be involved in osmotic pressure regulation in oysters.

*ChBADH*, *ChCDH*, and *ChBHMT* are the key enzymes involved in the synthesis and decomposition of betaine in oysters. *ChBADH* and *ChCDH* levels were high from January to April and from January to February, respectively. Other researchers have proposed that when oysters are subjected to external salinity stress, the expression levels of *ChBADH* and *ChCDH* increase, and betaine content increases accordingly [36]. *ChCDH* only remained at a high level from January to February, possibly because of its role as a pre-catalytic enzyme in the betaine synthesis pathway, and because its expression time was earlier than that of *ChBADH* [20]. The expression level of *ChBHMT* was high in January, possibly because of the continuous increase in salinity in the Maowei Sea of Guangxi in that mongth. Under high salt stress, *ChBHMT* expression decreases in oysters to maintain the corresponding osmotic pressure. In recent years, BHMT has been reported to maintain the osmotic pressure balance in various aquatic animals such as *Apostichopus japonicus* [37] and *Atlantic salmon* [38]. In our study, taurine content was the highest in April, and the expression of *ChCDO* and *ChCSAD* reached their highest levels in March and April, respectively. At this time, salinity was also at its highest level, indicating that oysters can improve the efficiency of taurine synthesis and adaptively compensate for high osmotic pressure. This indirectly suggests that *ChCDO* and *ChCSAD* are involved in the regulation of osmotic pressure in *C. hongkongensis* [39]. The expression of *ChCDO* peaked before that of *ChCSAD*, which may be because the former is an upstream regulatory factor in the taurine synthesis pathway.

### Coding region SNPs and development efficiency

Approximately 90% of the mutations in DNA sequences are single-base mutations, which contribute to the rich genetic diversity of organisms [40]. Genetic diversity is beneficial for the environmental adaptability and selective breeding of species. Most SNPs are located in the non-coding regions of the genome and play important roles in population genetics and evolutionary research [41, 42], whereas SNP loci in the coding region have high genetic stability [43]. In this study, 107 SNP loci were detected in five key gene-coding regions of *C. hongkongensis*, and 47 SNP markers were successfully developed using FLDAS-PCR and polyacrylamide gel technology. The success rate of SNP development was 43.9% (47/107). The success rate of SNP development using high resolution melting (HRM) technology for *C. gigas* glycogen phosphorylase was 33.3% (14/42) [44], reflecting the relatively high success rate of FLDAS-PCR technology in SNP development. However, FLDAS-PCR technology has low throughput and is not suitable for large-scale SNP detection and development.

### Correlations between SNPs and trait content

SNP sites with synonymous mutations do not alter the encoded amino acid sequence, but synonymous mutations can affect phenotypes by altering mRNA secondary structure stability, translation efficiency, and protein folding [45]. Non-synonymous mutations are single-nucleotide mutations that cause changes in amino acid sequences and that are likely to affect DNA transcription and subsequent translation processes, thereby affecting the structure and function of proteins. These are known as functional SNPs [46]. In this study, we identified nine SNP sites associated with taurine content in the *ChCDO* coding region of *C. hongkongensis*. *ChCDO*-46 and *ChCDO*-394 are non-synonymous mutations that cause amino acids changes from lysine to glutamate and from threonine to serine, respectively. Only one SNP, *ChCDH*-1585, was found to be associated with betaine content in the *ChCDH* coding region of *C. hongkongensis*. This is a non-synonymous mutation that causes an asparagine-to-aspartate change. Haploid-level analysis is considered more robust than single-marker allele-level analysis [47], and haplotype linkage disequilibrium and taurine association analyses can better illustrate the correlation between SNPs and taurine content. The results of this study showed that the five SNP loci that were significantly associated with taurine content formed a linkage group and resulted in three haplotypes, among which the GTACA haplotype had a significant advantage in taurine content.

## Conclusions

In this study, we cloned the full-length cDNA of *ChCDO* and *ChCSAD*, which are key genes involved in taurine metabolism in *C. hongkongensis*, and found that betaine and taurine contents and the expression of key genes were regulated by seawater salinity. Using Sanger sequencing and multiple sequence alignment, 107 candidate SNPs were predicted in the coding regions of the key genes involved in betaine and taurine metabolism in *C. hongkongensis*, including *ChCDH*, *ChBADH*, *ChBHMT*, *ChCDO*, and *ChCSAD*. Forty-seven SNPs were successfully validated and genotyped using FLDAS-PCR and gene fragment resequencing. Nine SNPs were associated with taurine content in the *ChCDO* coding region (*P* < 0.05), and one SNP was associated with betaine content in the *ChCDH* coding region (*P* < 0.05). The results of linkage disequilibrium analysis showed that the five SNPs in the coding region of *ChCDO* form a linkage group and result in three haplotypes, among which the haplotype GTACA has a significant advantage. These loci and haplotypes can serve as potential molecular markers for the molecular breeding of *C. hongkongensis*.

## Supplementary Material

Supplement 1

## Figures and Tables

**Table 1:** Primers used for full-length cDNA cloning and quantitative PCR

Primers	Primer sequences (5′-3′)	usages
15 CSAD-F1	ATGTGAGGGGTTCAAAGGTCAAAAAG	Fragment cloning
1433 CSAD-R1	TCATCATTCCCTCCTTGACCTTTGGC	Fragment cloning
430 CSAD-F2	GGTTTATGAAGGCTCGTGACCGCAA	Fragment cloning
1643 CSAD-R2	TCATCATTCCCTCCTTGACCTTTGGC	Fragment cloning
193 CDO-F1	TTTGAGGAAGGAGCTGGGCAATGGT	Fragment cloning
785 CDO-R1	ACATGAGATTCCAAGCAGGTCCATAA	Fragment cloning
397 CDO-F2	CATACACAACCACCCGAACTCACAC	Fragment cloning
699 CDO-R2	ACCACTGAAGTCCTGATCGAATCCCA	Fragment cloning
193 CDO-5-R1	ACCATTGCCCAGCTCCTTCCTCAAA	5’race
356 CDO-5-R2	CGTTCCAGCACAGAATCATCAGGTT	5’race
423 CDO-5-R3	ACGCCCTCTAGTGTTTTCATGAAGC	5’race
668 CDO-3-F1	GACGACACAAGCAAAGAGACGGAGG	3’race
627 CDO-3-F2	ATCTCTACTCGCCACCATTCACCAC	3’race
577 CDO-3-F3	CGTGCACAGAGTTGGAAACAGAAGT	3’race
433 CSAD-5-R1	TCATTGCGGTCACGAGCCTTCATAA	5’race
473 CSAD-5-R2	CTGCTTTAGTTCTTCGGGATAGGAG	5’race
685 CSAD-5-R3	AGGGAGAATACAGGGGACACTTCGT	5’race
1455 CSAD-3-F1	GGCTTCGAGAGAGACATTGACAATC	3’race
1391 CSAD-3-F2	TCAGTGTGGCCGCAAGAATGACGTT	3’race
932 CSAD-3-F3	TATGGGAATGGGCCTTGACAACCTC	3’race
M13F	TGTAAAACGACGGCCAGT	universal primer
M13R	CAGGAAACAGCTATGACC	universal primer
UPM	CTAATACGACTCACTATAGGGCAAGCAGTGGTATCAACGCAGAGT	RACE
UPS	CTAATACGACTCACTATAGGGC	RACE
400 qPCR-CDO-F1	GGAGCTCTACAGGAAATCGGA	RT-qPCR
573qPCR-CDO-R1	TGGTGAATGGTGGCGAGTAGA	RT-qPCR
137qPCR-CSAD-F	AAGCCCACAGAGAAGCAGAGAA	RT-qPCR
345qPCR-CSAD-R	CATTGCGGTCACGAGCCTTCAT	RT-qPCR
CDH-qRT-F	CCATCAGGCCACAACACTCTCTT	RT-qPCR
CDH-qRT-R	ACGCCGATCTTCTTCATCAC	RT-qPCR
BADH-qRT-F	CAGTCCTTGTATCCTCGTCAACT	RT-qPCR
BADH-qRT-R	TATTCCTCCTGCCAGTCCTAGTT	RT-qPCR
BHMT-qRT-F	TGTATTGGCTTTCACCTATTGC	RT-qPCR
BHMT-qRT-R	CGACGGCCCACTCTATCATTT	RT-qPCR
LActinF	CTGTGCTACGTTGCCCTGGACTT	RT-qPCR
LActinR	TGGGCACCTGAATCGCTCGTT	RT-qPCR

**Table 2:** SNPs associated with taurine and betaine contents

Number	SNPs	Genotype	Content (μg/g)	*P*-value	Associated trait
1	*ChCDO*-46	AA	873.16	0.004	taurine
		AG	843.39		
2	*ChCDO*-252	AA	872.15	0.010	taurine
		AG	845.32		
3	*ChCDO*-375	CC	873.45	0.027	taurine
		CT	845.78		
		TT	846.66		
4	*ChCDO*-384	GG	872.37	0.025	taurine
		GT	844.78		
		TT	882.99		
5	*ChCDO*-387	AA	872.76		
		AG	846.31	0.038	taurine
		GG	846.66		
6	*ChCDO*-393	CC	872.76	0.038	taurine
		CT	846.31		
		TT	846.66		
7	*ChCDO*-394	AA	872.34	0.029	taurine
		AT	844.17		
		TT	872.43		
8	*ChCDO*-417	CC	872.34	0.009	taurine
		CT	843.34		
9	*ChCDO*-423	AA	872.34	0.017	taurine
		AG	843.34		
		GG	930.72		
10	*ChCDH*-1585	G/G	905.48	0.017	Betaine
		AG	838.26		

**Table 3:** Information on non-synonymous mutation SNPs

SNPs	Nucleotide variant	Amino acid variant
*ChCDO*-46	A→G	Lys→Glu
*ChCDO-394*	A→T	Thr→Ser
*ChCDH-1585*	A→G	Asn→Asp

**Table 4:** Results of the haplotype analysis

Haplotype	CDO 387	CDO 393	CDO 394	CDO 417	CDO 423	Frequency	Taurine content (μg/g)
H1	A	C	A	C	A	0.781	873.88
H2	G	T	T	T	G	0.176	838.99
H3	G	T	A	C	A	0.033	930.72

## Data Availability

All data generated during this study or analysis are included in the published articles, sequence data is available in the NCBI GenBank database (https://www.ncbi.nlm.nih.gov/genbank/) under accession number OP792983 and OP792984.

## Abbreviations

ANOVA: analysis of variance
BADH: betaine aldehyde dehydrogenase
BHMT: betaine homocysteine methyltransferase
CDH: choline dehydrogenase
CDO: cysteine dioxygenase
CSA: cysteine sulfite
CSAD: cysteine sulfinic acid decarboxylase
ELISA: enzyme-linked immunosorbent assay
FLDAS-PCR: fragment length discrepant allele specific PCR
HRM: high resolution melting
ORF: open reading frame
UTR: untranslated region
