# Supplementary material for: Development of single nucleotide polymorphisms in key genes of taurine and betaine metabolism in Crassostrea hongkongensis and their association with content-related traits
Source: Res Sq. 2024 Dec 18:rs.3.rs-5097219. Preprint. [Version 1] doi: 10.21203/rs.3.rs-5097219/v1 (PMC11702833; doi:10.21203/rs.3.rs-5097219/v1)
Supplement: Supplement 1 [file NIHPPRS5097219v1-supplement-1.pdf]

Supplementary Files

This is a list of supplementary files associated with this preprint. Click to download.

- Additionalfile1.zip
- Additionalfile2.xlsx
- Graphicalabstract.pdf
